# Controlled Epitaxial Growth and Atomically Sharp Interface of Graphene/Ferromagnetic Heterostructure via Ambient Pressure Chemical Vapor Deposition

**DOI:** 10.3390/nano11113112

**Published:** 2021-11-18

**Authors:** Ruinan Wu, Yueguo Hu, Peisen Li, Junping Peng, Jiafei Hu, Ming Yang, Dixiang Chen, Yanrui Guo, Qi Zhang, Xiangnan Xie, Jiayu Dai, Weicheng Qiu, Guang Wang, Mengchun Pan

**Affiliations:** 1 College of Intelligence Science and Technology, National University of Defense Technology, Changsha 410073, China; a476658540a@163.com (R.W.); huyueguo1991@163.com (Y.H.); lips13@163.com (P.L.); junpingpeng@126.com (J.P.); garfield_nudt@163.com (J.H.); chendixiang@163.com (D.C.); guoyanrui_nudt@163.com (Y.G.); 13141317443@163.com (Q.Z.); 2 Department of Physics, College of Liberal Arts and Sciences, National University of Defense Technology, Changsha 410073, China; wjsmuzi@126.com (M.Y.); jydai@nudt.edu.cn (J.D.); 3 State Key Laboratory of High Performance Computing, College of Computer Science and Technology, National University of Defense Technology, Changsha 410073, China; xnx@mail.ustc.edu.cn; 4 State Key Laboratory of Low-Dimensional Quantum Physics, Department of Physics, Tsinghua University, Beijing 100084, China

**Keywords:** graphene, monolayer, single-crystal, heterostructure, epitaxial growth, ambient pressure chemical vapor deposition (APCVD)

## Abstract

The strong spin filtering effect can be produced by C-Ni atomic orbital hybridization in lattice-matched graphene/Ni (111) heterostructures, which provides an ideal platform to improve the tunnel magnetoresistance (TMR) of magnetic tunnel junctions (MTJs). However, large-area, high-quality graphene/ferromagnetic epitaxial interfaces are mainly limited by the single-crystal size of the Ni (111) substrate and well-oriented graphene domains. In this work, based on the preparation of a 2-inch single-crystal Ni (111) film on an Al_2_O_3_ (0001) wafer, we successfully achieve the production of a full-coverage, high-quality graphene monolayer on a Ni (111) substrate with an atomically sharp interface via ambient pressure chemical vapor deposition (APCVD). The high crystallinity and strong coupling of the well-oriented epitaxial graphene/Ni (111) interface are systematically investigated and carefully demonstrated. Through the analysis of the growth model, it is shown that the oriented growth induced by the Ni (111) crystal, the optimized graphene nucleation and the subsurface carbon density jointly contribute to the resulting high-quality graphene/Ni (111) heterostructure. Our work provides a convenient approach for the controllable fabrication of a large-area homogeneous graphene/ferromagnetic interface, which would benefit interface engineering of graphene-based MTJs and future chip-level 2D spintronic applications.

## 1. Introduction

The long spin-relaxation length and strong spin filtering effect have proven that graphene is an emerging material for two-dimensional (2D) spintronics [1,2,3,4,5]. The strong spin filtering effect at the lattice-matched graphene/Ni (111) interface has been theoretically predicted and experimentally studied [6,7] and results in an extreme TMR in vertical graphene/ferromagnetic (FM) spintronic devices [8,9,10]. However, the performance of graphene-based spintronic devices largely depends on the quality of the graphene/FM interface, and it is indeed a great challenge to controllably achieve large-size homogenous graphene/FM heterostructures with a well-oriented interface.

Epitaxially grown graphene (EGG) on single-crystal ferromagnetic metals via chemical vapor deposition (CVD) has been widely reported. E. D. Cobas [11] and A. Dahal [12] synthetized uncontrolled multilayer graphene on single-crystal NiFe (111) and Ni (111)/Y_2_O_3_ (111) substrates, respectively. However, it is still difficult to control the uniformity and orientation of graphene synthetized on ferromagnetic metal, as it is limited by the size of the single crystal substrate and the high solubility of carbon [11,12,13,14,15]. The segregation of carbon atoms at Ni grain boundaries easily results in the uneven growth of multilayer graphene, so that it fails to be seamlessly stitched into the intact single-crystal graphene film [16,17]. Plenty of studies on the epitaxial growth of single-layer graphene over Ni (111) film using strict ultra-high vacuum (UHV) CVD systems at low temperatures have been conducted to solve the problem of dissolved carbon [18,19,20]. However, it has a low efficiency, and the exploration of graphene grown on Ni (111) still requires wafer-scale Ni (111) films for EGG/FM heterostructures to be further improved.

Here, an efficient method has been developed to synthesize large-size uniform and wrinkle-free monolayer graphene by ambient pressure chemical vapor deposition (APCVD) based on the preparation technology of wafer-scale Ni (111) substrate. The graphene nucleation and subsurface carbon density are fine-tuned by the growth temperature and post-growth annealing to control the uniformity of EEG. The results of various characterizations indicate the high epitaxial quality of continuous EGG and the strong interfacial atomic coupling of a well-oriented graphene/Ni (111) interface. Our work lays the foundation for the high-efficiency production of high-quality EGG/FM heterostructures and serves to increase the applications of graphene-based spintronic devices.

## 2. Materials and Methods

The graphene is grown on Ni (111) films that are prepared by electron beam evaporation of Ni onto an α-Al_2_O_3_ (0001) wafer (Kejing, Anhui Province, China, size of 2 inches, thickness of 0.5 mm). Wafer-scale single-crystal Ni (111) (self provided) substrate is the most important prerequisite for the oriented growth of an ultra-flat graphene film. Firstly, the 2-inch α-Al_2_O_3_ (0001) wafer is annealed at high temperature in a quartz tube with an oxygen atmosphere to obtain an atomically smooth and impurity-free surface, as previously reported [21]. Secondly, a Ni film with 300 nm thickness is deposited at 480 °C with a background pressure of 5.0 × 10^−4^ Pa and a deposition rate of 0.2 nm/s for the initial (111) oriented growth of the Ni film. Finally, the Ni film is annealed in the CVD system at 950 °C under ambient pressure with an H_2_/Ar mixture of 10 sccm/50 sccm to improve the crystallinity and catalytic activity of the Ni (111) substrate.

The graphene growth process is shown in Supplementary Figure S1. After the Ni (111) annealing process, CH_4_ is introduced into the chamber at a set of growth temperatures for adjusting the nucleation density of the graphene. It is proven that the nucleation density of graphene decreases with increasing growth temperature due to increases in the carbon solubility [22] and the desorption rate [23]. Then, it is followed by the post-growth annealing process without the supply of CH_4_ and H_2_ for critically regulating the subsurface carbon density. Finally, well-oriented graphene domains merge seamlessly to form a full-covered EGG on the Ni (111) surface during the segregation process of carbon, when the system is rapidly cooled down by slipping away the furnace.

## 3. Results and Discussion

**Figure 1:**
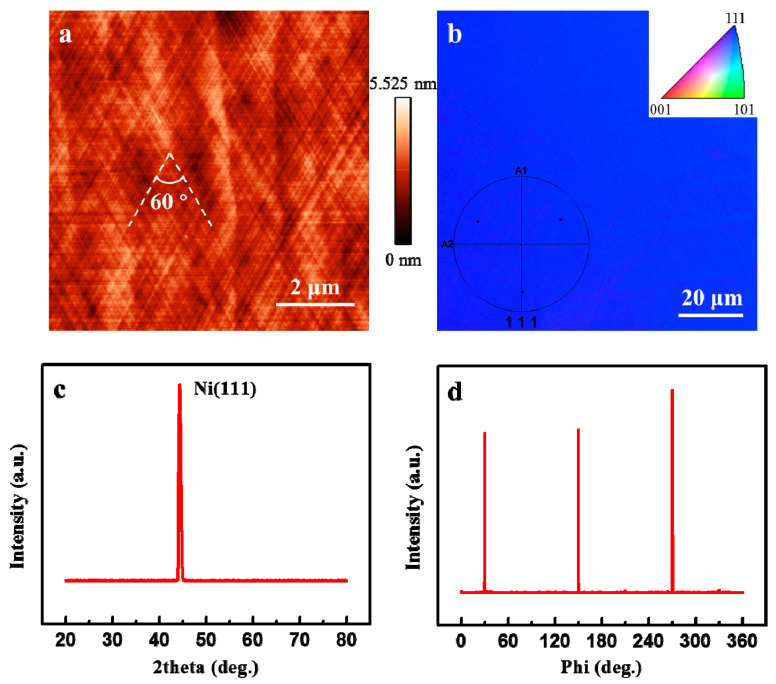
Typical characterization results of the single-crystal Ni (111) film. (**a**) AFM image of the Ni (111) film annealed in the quartz tube. (**b**) Out-of-plane EBSD mapping of the Ni (111) thin film. The inset shows the pole figure of the same region. (**c**) XRD θ-2θ scan and (**d**) HRXRD azimuthal off-axis φ scan of the Ni (111) film.

Figure 1a shows a typical atomic force microscope (AFM, NT-MDT TS-150, Moscow, Russia) image of Ni (111) films after the annealing process in the APCVD system. Obviously, we finally obtain an ultra-flat and clean surface morphology with a roughness of only 0.26 nm. Meanwhile, the clear slip lines with an angle of 60° (marked as the white line in Figure 1a) show textures of Ni film along three orientations at an angle of 60°, which is considered to be the result of the threefold symmetry of the metal films with the energetically favorable out-of-plane (111) orientation [21,24]. Additionally, the electron backscatter diffraction (EBSD, ULVAC PHI-710, Kanagawa, Japan) mapping (see Figure 1b) in the uniform blue reveals the out-of-plane (111) orientation without the twin structures combined with the four evenly distributed points in the pole figure (inside of Figure 1b). This is furtherly demonstrated by the X-ray diffraction (XRD, Bruker D8 discover, Germany) results. As shown in Figure 1c, it has only one sharp peak at 44.6° in the θ–2θ curves, suggesting the out-of-plane orientation of (111) over the whole Ni film. As for the in-plane orientation characterization, the azimuthal off-axis φ scan is carried out by rotating the sample normal to its surface with the high-resolution XRD (HRXRD, Bruker D8 discover, Bremen, Germany). The diffraction peaks of Ni film at an interval of 120°, as shown in Figure 1d, indicate the threefold symmetry of the Ni film, and thus the single-crystal nature is testified.

**Figure 2:**
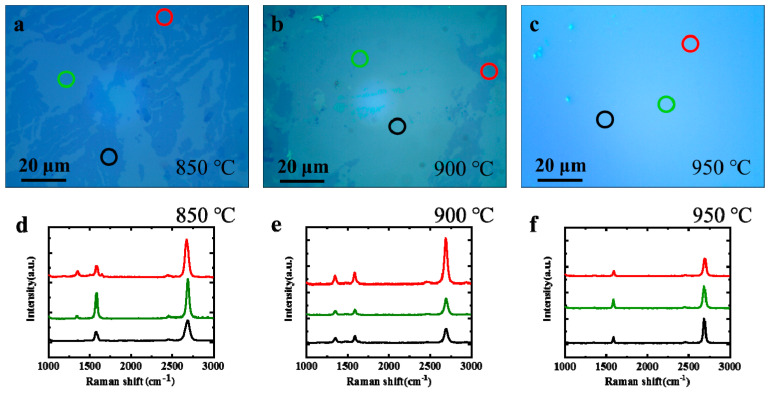
Characterization of graphene obtained at different temperatures. (**a**–**c**) OM images of transferred graphene on SiO_2_ substrate at 850, 900 and 950 °C, respectively. (**d**–**f**) Raman spectra of as-grown graphene with different thicknesses at different temperatures. Raman curves correspond to the regions in (**a**–**c**) marked as circles with the same color as themselves.

The growth of graphene on Ni (111) is a complex heterogeneous catalytic reaction. Normally, it contains two fundamental paths, i.e., direct catalytic growth on the Ni surface and circumlocutory segregation growth below the Ni surface [15]. To uncover the effects of temperature on these two growth paths, a series of growth temperatures have been investigated systematically. Figure 2a–c shows the typical optical microscope (OM, Nikon LV150NL, Tokyo, Japan) morphologies of the transferred graphene on SiO_2_ substrates of 500 nm, which are synthesized on Ni (111) films at different temperatures. Different graphene layers are identified through the comparison of different gray levels. The light transmittance of graphene decreases with an increase in layers. At 850 °C, many discontinuous bilayer graphene pieces are clearly observed in Figure 2a. The layers and defects of graphene are further characterized by Raman spectra (Horiba LABRAM HR, Kyoto, Japan), as shown in Figure 2d. This demonstrates that the monolayer graphene in the bright region has an obvious defect in the D band (1350 cm^−1^). On the other hand, the 2D/G band ratio of the graphene in the deep region is even higher than that of the monolayer graphene, as shown in Figure 2d. This indicates a non-AB stacked bilayer without electronic coupling [25]. Meanwhile, the torsion angle of the bilayer graphene is further confirmed by selected area electron diffraction (SAED, FEI Tecnai G2 F20 S-TWIN, USA) (see Figure S2). At 950 °C, the 2D/G band ratio of the Raman spectra in the lighter gray region is about 3, indicating a typical monolayer graphene film with no obvious defects. A transmission electron microscope (TEM, FEI Tecnai G2 F20 S-TWIN, USA) is used to confirm the monolayer graphene layer grown at 950 °C, as shown in Figure S3. In short, the intensity of the defective D band in the monolayer graphene decreases with the growth temperature, which indicates that the monolayer graphene quality improves as the temperature is increased.

**Figure 3:**
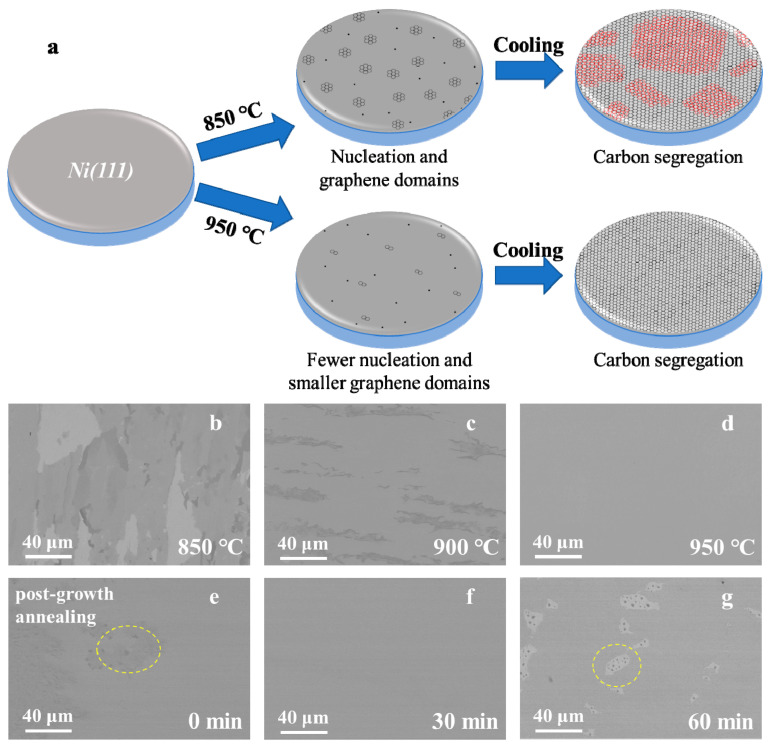
Regulating the growth process of graphene for single-crystal monolayer graphene. (**a**) Schematics for the fabrication of graphene on Ni (111) film at two topical temperatures, 850 and 950 °C, respectively. (**b**–**d**) Typical SEM images of graphene grown on Ni (111) film at 850, 900 and 950 °C, respectively. (**e**–**g**) Typical SEM images of graphene grown on Ni (111) film under different post-growth annealing times at 950 °C.

Generally, there is inevitable damage to and residual adhesive on graphene films during the process of transferring the graphene onto the SiO_2_ substrate [26], and the evaluation of the wide range of thickness uniformity is seriously affected. Hence, the typical images of the graphene on Ni (111) film obtained at different temperatures are directly characterized by scanning electron microscope (SEM, FEI Quanta FEG 250, USA) in Figure 3b–d. These images both show that the thickness uniformity of the graphene layer is improved gradually with the growth temperature. Combined with the Raman spectra and OM images of the transferred graphene on the SiO_2_ substrate (RDMICRO, Jiangsu Province, China, (100), resistivity: 2–4 mΩ·cm), the non-uniform distribution of graphene with the main characteristics of monolayer and bilayer structure types at 850 °C is furtherly confirmed (see Figure 3b). It is important to note that the monolayer graphene could be accurately prepared at 950 °C and fully cover the Ni surface (see Figure 3d), which demonstrates the perfect uniformity and high quality of large-scale graphene. The additional regions in samples at 850, 900 and 950 °C are characterized by SEM in detail, as shown in Figures S4–S7, respectively.

A reasonable physical model of graphene growth with different temperatures has been established, as shown in Figure 3a. According to the classical two-dimensional nucleation theory, a low temperature is conducive to the adsorption of carbon species on Ni (111) surface, and the graphene nucleation density decreases with increasing growth temperature [27]. At 950 °C, few nucleation sites and small graphene domains form on Ni (111) surface at the preliminary stage. Then, the graphene domains with the same orientation merge seamlessly to form a single-crystal graphene covering the whole Ni (111) surface by adopting the segregated carbon atoms during the rapid cooling process. More regions in samples at 950 °C are characterized by Raman in detail, as shown in the Figure S8. By contrast, more nucleation sites and larger graphene domains formed on the Ni (111) surface with the growth temperature decreased from 950 to 850 °C. Then, while the first layer graphene domains rapidly expand and cover the Ni (111) surface, the second layer may subsequently grow by cutting into the interface between the first layer and Ni (111) substrate in the segregation process of carbon atoms [22]. The second layer graphene, whose orientation may not be unique, is marked in red in Figure 3a. As a result, a mixed morphology of monolayer and bilayer graphene could be obtained at 850 °C, as shown in Figure 3b. The analysis shows that the initial graphene nucleation density is an important factor in growing uniform monolayer graphene [20].

It should be noted that the post-growth annealing time greatly affects the uniformity and integrity of monolayer graphene. There are plenty of nonuniform thick graphene spots marked as yellow dotted circles in Figure 3e. After the introduction of the post-growth annealing process, the uniformity of graphene becomes better as the spots disappeared, as shown in Figure 3f. The main reason is that the subsurface carbon density is higher than the carbon density of the Ni (111) bulk, and the carbon atoms continuously diffuse into Ni (111) bulk during the process of post-growth annealing, which results in decreases in the subsurface carbon density. Low subsurface carbon density is beneficial to the formation of monolayer graphene on Ni (111), which is in agreement with the simulation results from the literature [28]. However, Figure 3g shows that there are some graphene holes with a post-growth annealing time of 60 min. The integrity becomes worse when the annealing time is too long, which is attributed to the shortage of subsurface carbon density. Above all, both graphene nucleation and subsurface carbon density contribute to the result of uniform monolayer graphene.

**Figure 4:**
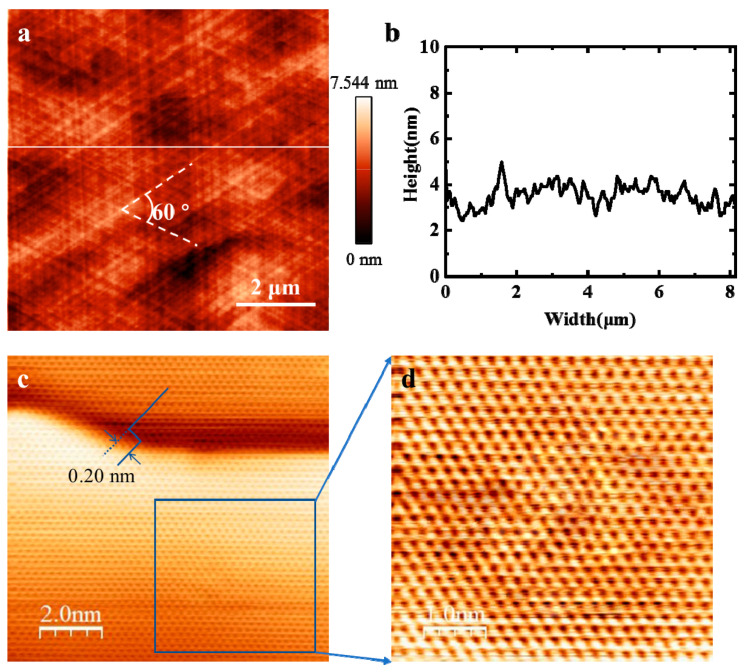
Microstructure characterization of graphene grown on Ni (111) film at 950 °C. (**a**) The AFM image of graphene grown on Ni (111) with an average roughness of 0.64 nm. (**b**) A profile of apparent height in the AFM image along the white line marked in (**a**). (**c**) STM image of graphene on the Ni (111) surface with an atomic metal step measured under a tunneling current of 1 nA and bias of 100 mV at room temperature. (**d**) High-resolution STM image of graphene on a Ni (111) terrace marked as the rectangular box in (**c**).

In order to analyze the quality of EGG grown on Ni (111) at 950 °C, characterizations of the atomic morphology, defects and flatness are employed for evaluating the microstructure of EGG. The topical AFM image of EGG is shown in Figure 4a. No obvious folds appear in the field of view, which benefits from the seamless stitching of graphene domains without producing grain boundaries. Besides, Figure 4b shows the height trace of the white line noted in Figure 4a. The maximum fluctuation of the atomically smooth surface is about 7.5 nm, and the average roughness is only 0.64 nm. Meanwhile, the obviously threefold symmetry step textures with the angle of 60° are clearly displayed in the graphene/Ni (111) sample, which is similar to the data of the Ni (111) film. This suggests the growth of graphene follows the terraces and steps of the Ni (111) surface. The atomic structure of as-grown EGG is characterized by scanning tunneling microscope (STM, Specs STM 150, Berlin, Germany) in detail, as shown in Figure 4c. The (1 × 1) graphene structure is formed based on the identical lattice constants of Ni (111) and graphene (2.49 Å for Ni (111) and 2.46 Å for graphene), mostly due to the tiny lattice mismatch of graphene with Ni (111) [29]. This indicates the high quality of our EGG sample. Moreover, the STM also shows that there are two atomic faces, as shown in Figure 4c, and the step height of 0.20 nm is approximately equal to the atomic layer thickness of the Ni (111) film, which is less than that of the graphene layer (about 0.34 nm) [20]. Therefore, the image reveals the information of two terraces in the Ni (111) film with one atom step. It is worth noting that the hexagonal lattice distribution of the graphene uninterruptedly extends across the step of the Ni (111) film without any wrinkles. Figure 4d shows the enlarged STM image of graphene on one terrace, as noted by the rectangular box. Remarkably, no defects or wrinkles are formed in the graphene film grown on terraces of the Ni (111) surface. The reasonable explanation for the formation of wrinkle-free graphene is primarily attributed to two factors: the strong interaction between the graphene and the substrate, and the low thermal expansion mismatch between the graphene and the substrate [24].

**Figure 5:**
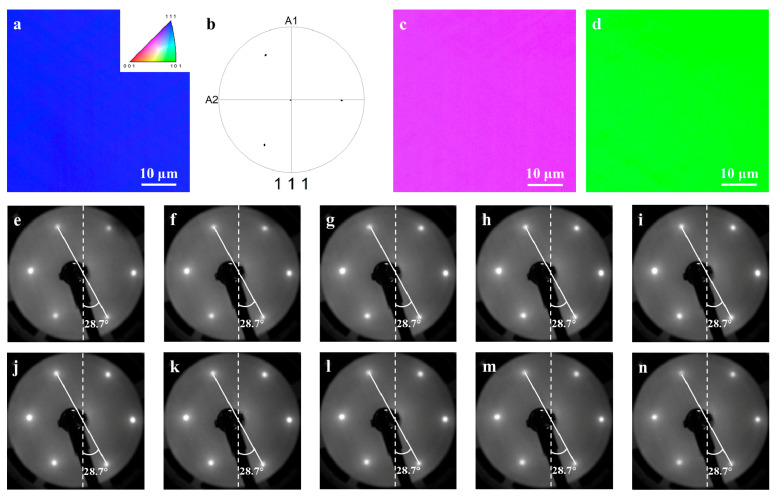
Characterization of the lattice orientation of the graphene/Ni (111) epitaxial interface obtained at 950 °C. (**a**) Out-of-plane EBSD mapping of the Ni (111) thin film after graphene growth. (**b**) The pole figure of the same region. (**c**) In-plane EBSD mapping of Ni (111) thin film after graphene growth in a random direction. (**d**) In-plane EBSD mapping of Ni (111) thin film in the vertical direction. (**e**–**n**) A series of LEED patterns of monolayer graphene synthesized on Ni (111) film at 950 °C are acquired with a step of 0.5 mm at a primary energy of 106.9 eV. The diameter of the measurement spots is 0.5 mm.

The EBSD mapping of EGG/Ni (111) film (see Figure 5a–d) is entirely identical to that of Ni (111) film, which reveals that the crystallinity of Ni (111) films is kept well during the process of graphene growth. To further characterize the epitaxial quality of the entire graphene/Ni (111) interface, low energy electron diffraction (LEED, BDL 600IR, Ontario, Canada) characterization is applied to provide evidence for crystal orientation of the monolayer graphene synthesized on Ni (111) film at 950 °C. Figure 5e–n provides an overview of LEED patterns along a straight line across the entire sample in 0.5 mm steps for evaluating the orientation distribution of EGG (supporting information is shown in Figure S9). It is noted that the hexagonal diffraction patterns of six spots with equal brightness is found in graphene/Ni (111) film. The formation of graphene could be identified from the behavior of the spot intensity. When there is no graphene on the Ni surface, as shown in Figure S10, the electron diffraction patterns of clean Ni (111) are three bright and three dark spots due to the three-fold rotational symmetry of Ni (111) [20,30,31]. By contrast, the diffraction patterns of graphene show six evenly distributed spots with equal brightness, owing to the six-fold rotational symmetry of graphene. As a result, it is proven that graphene does exist on the Ni (111) film, which concurs with the characterization of Raman and SEM. In addition, the relative angle between graphene and Ni (111) could be effectively extracted from the patterns. As shown in Figure 5e–n, the angles between the diagonal spots and the vertical direction (marked in the figures) in all of the LEED patterns are 28.7°, and no additional diffraction spots or rotation spots of graphene are observed. Therefore, it is inferred that the carbon atoms of the monolayer graphene are precisely above the Ni atoms, blocking the scattering of the Ni (111) film [32]. The results prove the large-scale crystallinity of the continuous graphene film, which consistently follows the orientation of the Ni (111) film [33].

**Figure 6:**
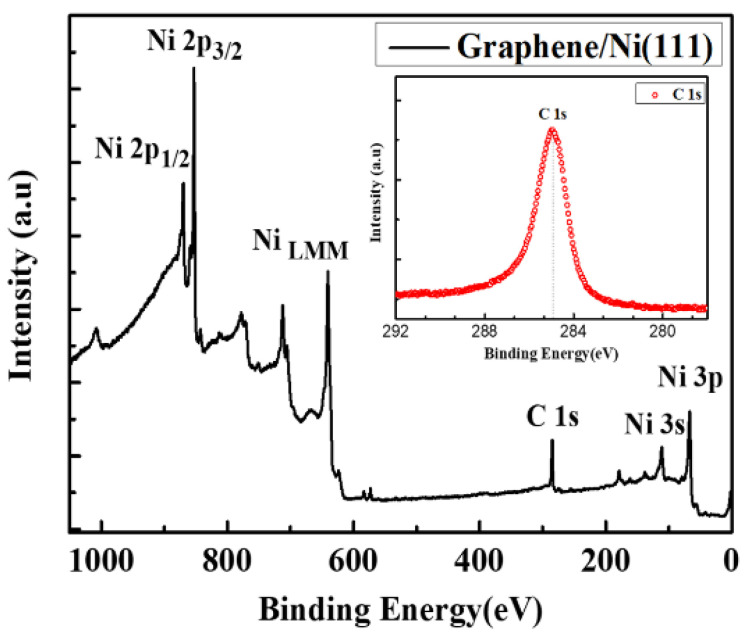
XPS spectra of the graphene/Ni (111) interface. The inset shows the C 1s peak of the graphene at 285.0 eV.

The coupling effect between EGG and Ni (111) substrate is further confirmed by X-ray photoelectron spectroscopy (XPS, Specs PHOIBOS 100, Berlin, Germany), as shown in Figure 6. The peaks observed in the XPS measures are only related to nickel and carbon elements, which shows no other impurities in the surface of the graphene/Ni (111) samples. More importantly, the distinct C 1s peak appeared after graphene growth, compared with XPS data of pure Ni (111) films reported in the literature [20,30]. Besides, the C 1s peak with a binding energy of 285.0 eV is higher than 284.4 eV in graphite, which shows a strong interaction between graphene and Ni (111). This is probably due to the orbital hybridization of the C and Ni atoms [30,34]. This strong orbital hybridization between graphene and Ni atoms is generally considered to be the main reason for the perfect spin filtering of the graphene/Ni (111) interface, as previously studied [35,36]. Hence, the full-covered, clean, epitaxial and strong coupling graphene/Ni (111) interface prepared by our method will be a highlight for graphene spintronics.

## 4. Conclusions

In summary, we have developed a robust and reliable APCVD strategy for controlled epitaxial growth of uniform monolayer graphene on single-crystal Ni (111) films with an atomically perfect graphene/FM interface. The optimal growth method is successfully achieved by making a tune of the well-oriented nucleation process of the Ni (111) catalytic surface and the segregation of the subsurface carbon atoms. High crystallinity and uniform morphology of EGG are systematically investigated, while well-oriented alignment and a strong coupling interaction with the lattice-matched Ni (111) film have been carefully identified, which are attributed to the fact that graphene can uninterruptedly extend across the step of Ni (111) film and the well-oriented domains seamlessly merge into a continuous EGG. Our work provides a feasible method for the efficient production of high-quality graphene/ferromagnetic heterostructures, which contributes to the development of future 2D spintronic applications.

## Data Availability

Not applicable.

## Supplementary Materials

The following are available online at https://www.mdpi.com/article/10.3390/nano11113112/s1, Figure S1: Schematic of graphene growth processes and the corresponding flow rates in each step of uniform monolayer graphene growth at 850–950 °C at ambient pressure. Figure S2: SAED patterns of (**a**) mono- and (**b**) bi-layer graphene obtained at 850 °C. Figure S3: TEM image of graphene grown on Ni (111) at 950 °C. Figure S4: Schematic of how the SEM images sequence is recorded across the sample. Figure S5: SEM images of graphene grown on Ni (111) at 850 °C for nine regions in Figure S4. Figure S6: SEM images of graphene grown on Ni (111) at 900 °C for nine regions in Figure S4. Figure S7: SEM images of the grown graphene in different regions at 950 °C. Figure S8: Raman spectra characterization of the sample. (**a**) the whole sample is divided into six regions (**b**) the Raman re-sults are performed in each region. Figure S9: The scanning LEED across the entire sample. (**a**) Schematic of how the LEED patterns sequence is recorded across the sample. (**b**) LEED patterns sequences taken across the entire width of the sample in 0.5 mm steps. Figure S10: LEED patterns of a clean Ni (111) film and graphene synthesized on Ni (111) film. The LEED patterns are recorded for a primary electron energy of 106.9 eV. The diameter of the measurement spots is 0.5 mm.
