# Supplementary material for: Controlled Epitaxial Growth and Atomically Sharp Interface of Graphene/Ferromagnetic Heterostructure via Ambient Pressure Chemical Vapor Deposition
Source: Nanomaterials (Basel). 2021 Nov 18;11(11):3112. doi: 10.3390/nano11113112 (PMC8620976; doi:10.3390/nano11113112)
Supplement: Supplementary file 1 [file nanomaterials-11-03112-s001.zip › nanomaterials-1388887 SI 2ndproof-revised.pdf]

nanomaterials

Supporting Information

Controlled Epitaxial Growth and Atomically Sharp Interface of Graphene/Ferromagnetic Heterostructure via Ambient Pressure Chemical Vapor Deposition

Ruinan Wu [1], Yueguo Hu [1], Peisen Li [1], Junping Peng [1], Jiafei Hu [1], Ming Yang [2], Dixiang Chen [1], Yanrui Guo [1], Qi Zhang [1], Xiangnan Xie [3], Jiayu Dai [2], Weicheng Qiu [1,*], Guang Wang [2,4,*] and Mengchun Pan [1,*]

[1] College of Intelligence Science and Technology, National University of Defense Technology, Changsha 410073, China; a476658540a@163.com (R.W.); huyueguo1991@163.com (Y.H.); lips13@163.com (P.L.); junpingpeng@126.com (J.P.); garfield_nudt@163.com (J.H.); chendixiang@163.com (D.C.); guoyanrui_nudt@163.com (Y.G.); 13141317443@163.com (Q.Z.)
[2] Department of Physics, College of Liberal Arts and Sciences, National University of Defense Technology, Changsha 410073, China; wjsmuzi@126.com (M.Y.); jydai@nudt.edu.cn (J.D.)
[3] State Key Laboratory of High Performance Computing, College of Computer Science and Technology, National University of Defense Technology, Changsha 410073, China; xnx@mail.ustc.edu.cn
[4] State Key Laboratory of Low-Dimensional Quantum Physics, Department of Physics, Tsinghua University, Beijing 100084, China
* Correspondence: 2008qiuweicheng@sina.com (W.Q.); wangguang@nudt.edu.cn (G.W.); pmc_nudt@vip.163.com (M.P.)

Citation: Wu, R.; Hu, Y.; Li, P.; Peng, J.; Hu, J.; Yang, M.; Chen, D.; Guo, Y.; Zhang, Q.; Xie, X.; et al. Controlled Epitaxial Growth and Atomically Sharp Interface of Graphene/Ferromagnetic Heterostructure via Ambient Pressure Chemical Vapor Deposition. *Nanomaterials* **2021**, *11*, 3112. https://doi.org/10.3390/nano11113112

Received: 4 September 2021
Accepted: 13 November 2021
Published: 18 November 2021

Publisher's Note: MDPI stays neutral with regard to jurisdictional claims in published maps and institutional affiliations.

Copyright: © 2021 by the authors. Licensee MDPI, Basel, Switzerland. This article is an open access article distributed under the terms and conditions of the Creative Commons Attribution (CC BY) license (http://creativecommons.org/licenses/by/4.0/).

The APCVD Growth of Graphene on the Ni (111) Thin Film

Before the graphene growth, the Ni substrate is placed into the quartz tube and heated from room temperature to growth temperature of 850–950 °C during 45 min in a gas flow of H_2 (10 sccm) and Ar (50 sccm) to clean the surface oxidation. Then, the Ni substrate is annealed for another 60 min to improve the crystallinity and catalytic activity of the Ni (111) substrate. The CH_4 (1 sccm) is introduced into the chamber for 38 min and the temperature is kept at growth temperature, during the growth process. After that, the post-growth annealing process is taken without the supply of CH_4 and H_2 for 30 min. In this process, carbon atoms dissolve from Ni (111) surface into the bulk phase, and the concentration gradient of carbon in Ni (111) decreases over time. It is noted that the amount of carbon atoms near the surface decreases. Finally, the sample is rapidly cooled down by slipping away the furnace. The carbon atoms precipitate from the bulk under the effect of concentration gradient, due to the decrease of carbon solubility in Ni (111). After the above processes, well-oriented graphene domains merge seamlessly to form a full-covered single-crystal graphene film on Ni (111) surface.

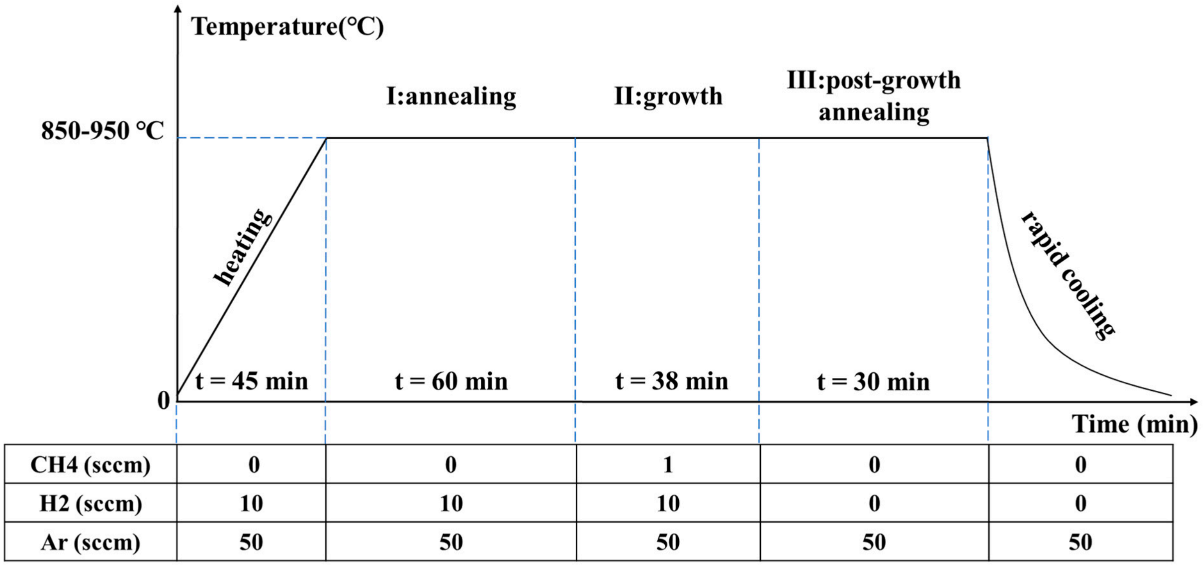

CH4 (sccm)	0	0	1	0	0
H2 (sccm)	10	10	10	0	0
Ar (sccm)	50	50	50	50	50

Figure S1. Schematic of graphene growth processes and the corresponding flow rates in each step of uniform monolayer graphene growth at 850–950 °C at ambient pressure.

Selected Area Electron Diffraction (SAED) and Transmission Electron Microscope (TEM) Measurement

The graphene sample is spin-coated with a polymethyl methacrylate (PMMA, Kejing, Anhui Province, China), and then heated to 150 °C to be stabilize. The PMMA/graphene/Ni (111)/Al_2O_3 sample is placed on a mixture of $FeCl_3$ (Zhanwang, Jiangsu Province, China, ≥99% purity) and HCl (Xingkonghuabo, Hunan Province, China, 36–38% purity) to corrode the Ni film from the edge, which intending to make sure that the PMMA/graphene film would float on the surface and not immerse in the liquor. The isolated PMMA/graphene film is cleaned twice with deionized water for 20 min per time, and then placed on a Cu grid. After dying naturally, the PMMA is removed with acetone (Fuyu Fine Chemical, Tianjin, China, ≥99.5% purity), and then cleaned with absolute alcohol (Jincheng, Jiangsu Province, China, ≥99.7% purity). SAED (FEI Tecnai G2 F20 S-TWIN, USA) is performed on the bilayer graphene islands and consecutive monolayer graphene film. Two sets of diffraction spots with rotation angle of 19.7° are exhibited in the region of bilayer graphene, indicating the structure of non-AB stacking, as shown in Figure S2b. In addition, TEM (FEI Tecnai G2 F20 S-TWIN, USA) is used to confirm graphene layer obtained at 950 °C. And graphene obtained on Ni (111) is a singer layer at 950 ℃, as shown in Figure S3.

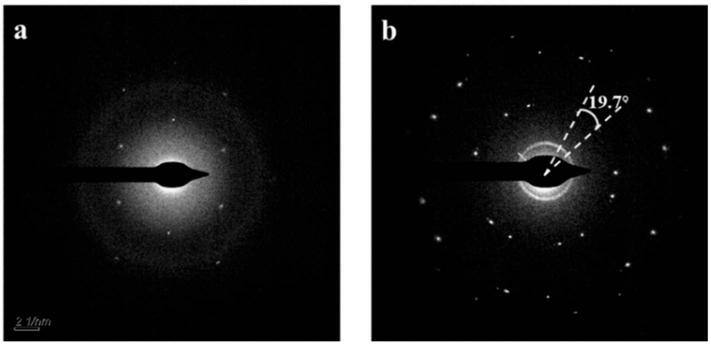

Figure S2. SAED patterns of (**a**) mono- and (**b**) bi-layer graphene obtained at 850 °C.

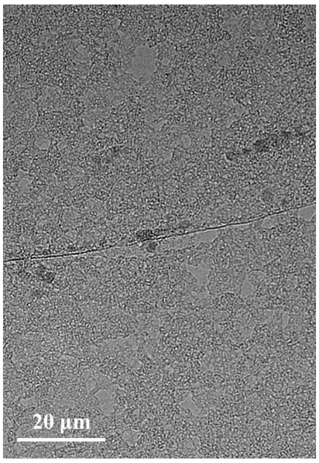

Figure S3. TEM image of graphene grown on Ni (111) at 950 °C.

SEM Characterization of Graphene Obtained at 850 and 900 °C

The graphene/Ni (111) sample is artificially divided into nine equal regions, as shown in Figure S4, and SEM (FEI Quanta FEG 250, USA) characterization is carried out in each region.

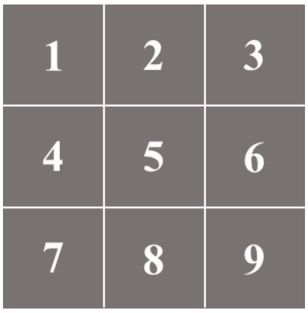

Figure S4. Schematic of how the SEM images sequence is recorded across the sample.

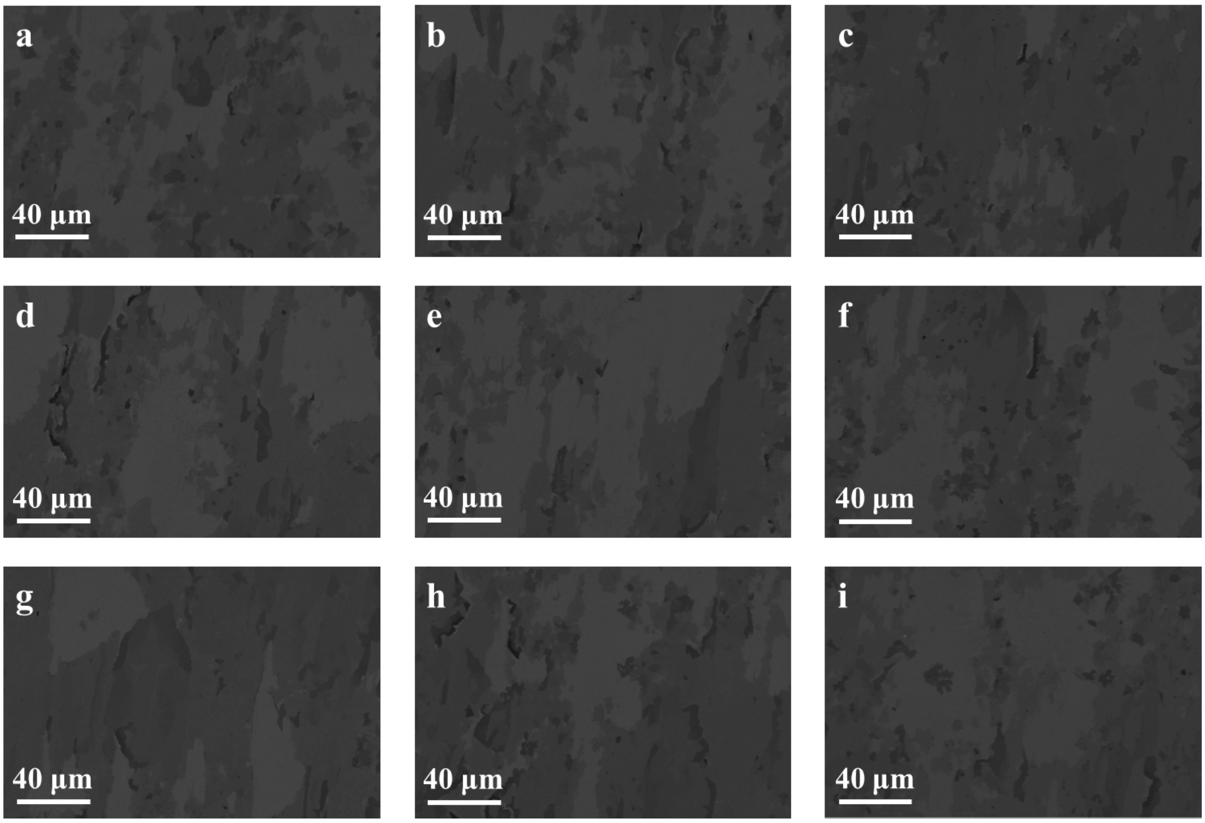

Figure S5. SEM images of graphene grown on Ni (111) at 850 °C for nine regions in Figure S4.

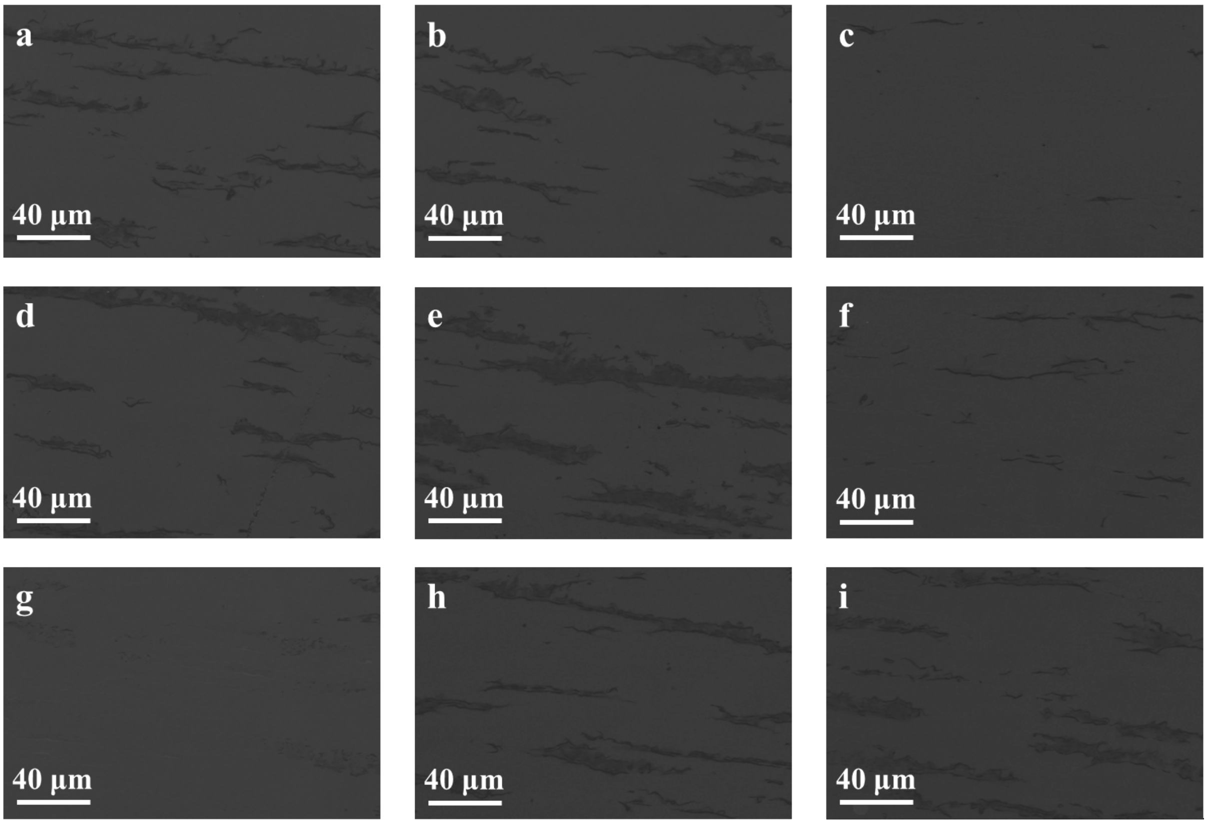

Figure S6. SEM images of graphene grown on Ni (111) at 900 °C for nine regions in Figure S4.

Large-Area Uniformity and Large-Scale Crystallinity of the Grown Graphene at 950 °C

In order to characterize large-area uniformity of the grown graphene at 950 °C, the graphene/Ni (111) sample is artificially divided into nine equal regions, as shown in Figure S4, and SEM characterization is carried out in each region. Through the statistics of multiple results, the layer thickness uniformity of the whole sample could be represented by SEM images in different regions. In Figure S7, all SEM images show only one gray, which proved a uniform graphene film obtained at 950 °C.

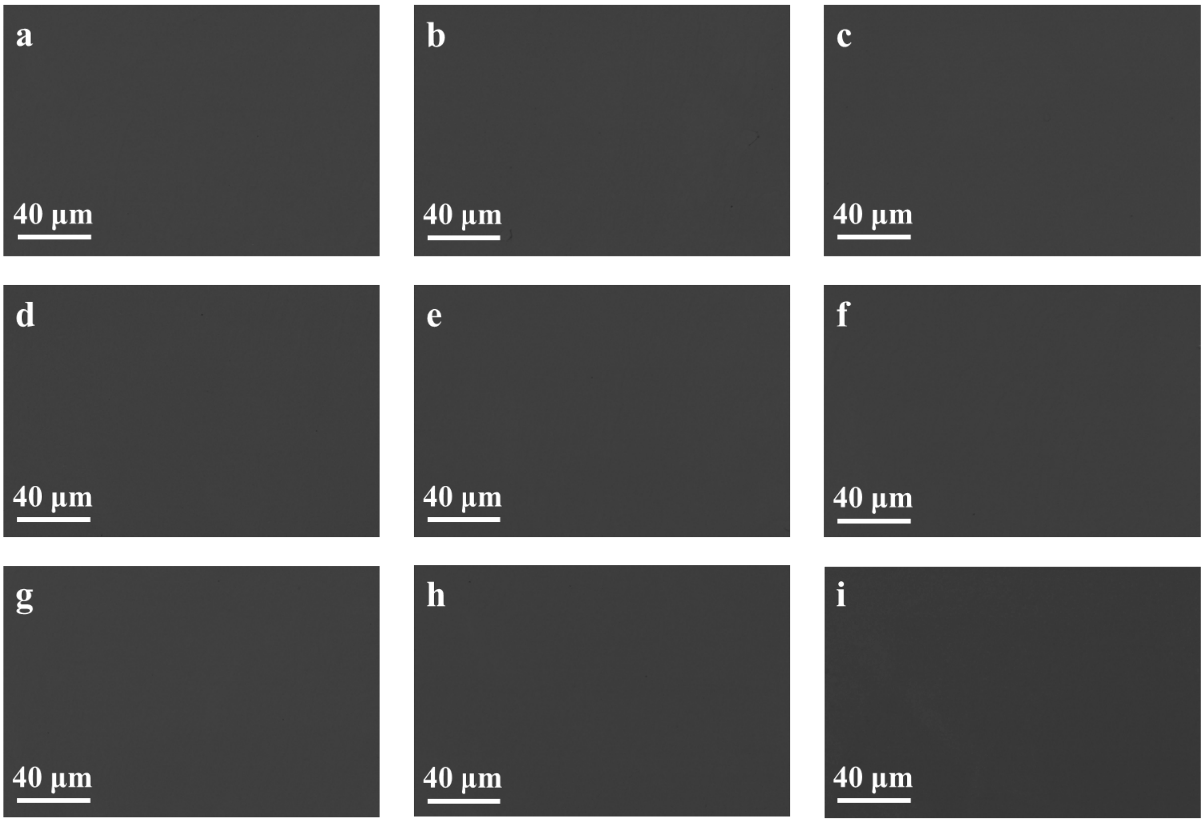

Figure S7. SEM images of the grown graphene in different regions at 950 °C.

Besides, the transferred graphene on SiO_2 substrate (RDMICRO, Jiangsu Province, China, (100), resistivity: 2–4 mΩ·cm) is artificially divided into six regions, and Raman spectra (Horiba LABRAM HR, Kyoto, Japan) is carried out in each region. The results are shown in Figure S8, all the 2D/G band ratios of the Raman spectra are about 3, and no obvious defect D band (1350 cm^{-1}) is observed, indicating the high quality and uniformity of monolayer graphene.

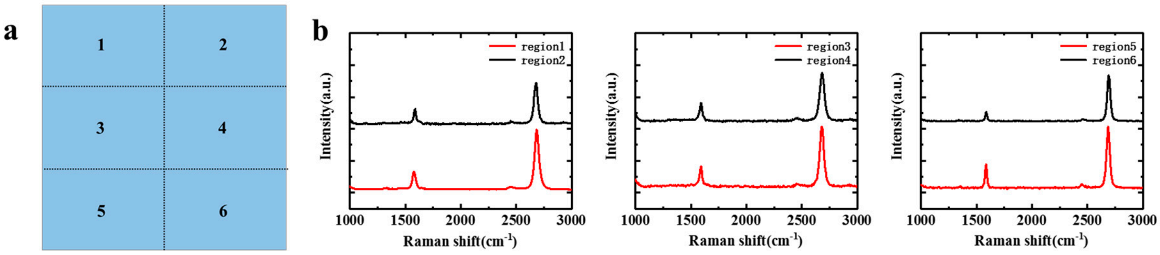

Figure S8. Raman spectra characterization of the sample. (**a**) the whole sample is divided into six regions (**b**) the Raman results are performed in each region.

In order to characterize large-scale crystallinity of the grown graphene at 950 °C, the scanning LEED (BDL 600IR, Ontario, Canada) image is taken across the entire width of the sample. The diameter of the measurement spots is 0.5 mm, and the scanning step is 0.5 mm. The results show that the angles between the diagonal spots and the vertical direction (marked in the figures) in all of the LEED patterns are 28.7°, and no additional diffraction spots or rotation spots of graphene are observed. The crystal orientation of graphene in different regions is consistent, which confirm the large-scale crystallinity of the entire graphene film.

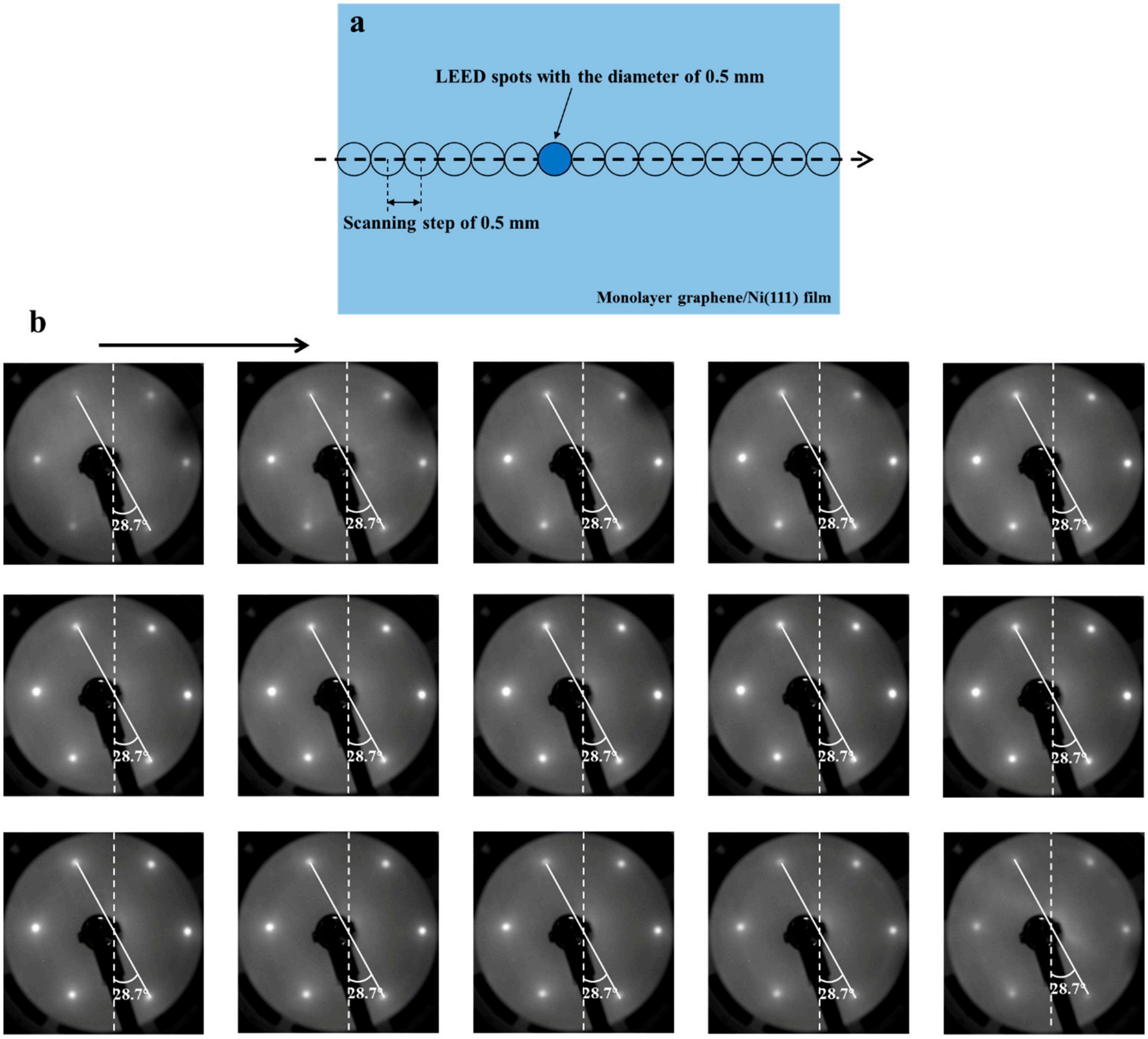

Figure S9. The scanning LEED across the entire sample. (**a**) Schematic of how the LEED patterns sequence is recorded across the sample. (**b**) LEED patterns sequences taken across the entire width of the sample in 0.5 mm steps.

LEED Comparison of Ni (111) and Monolayer Graphene on Ni (111)

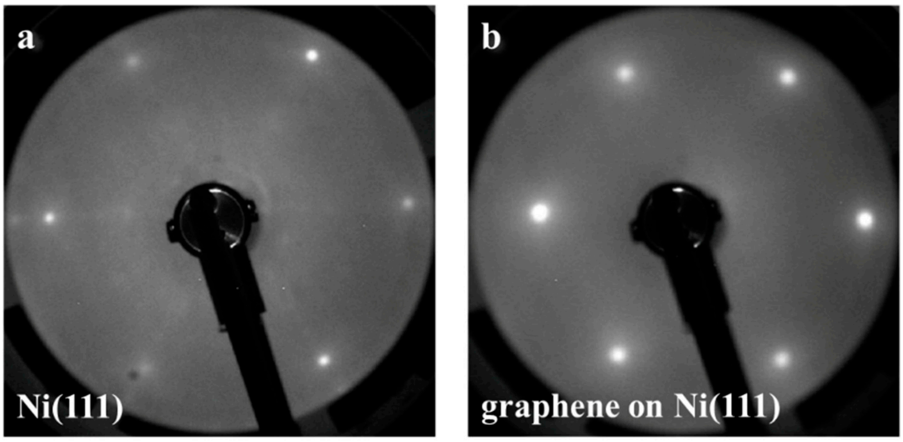

Figure S10. LEED patterns of (**a**) a clean Ni (111) film and (**b**) graphene synthesized on Ni (111) film. The LEED patterns are recorded for a primary electron energy of 106.9 eV. The diameter of the measurement spots is 0.5 mm.
